# Dynamics of the US Housing Market: A Quantal Response Statistical Equilibrium Approach

**DOI:** 10.3390/e20110831

**Published:** 2018-10-30

**Authors:** Özlem Ömer

**Affiliations:** Department of Economics, The New School for Social Research, 6 E 16th Street, New York, NY 10011, USA; omero679@newschool.edu; Tel.: +90-542-723-8050

**Keywords:** housing market crash, statistical equilibrium, quantal response, informational entropy, the maximum entropy method, C18, D89, D90, E30, G01, R39

## Abstract

In this article, we demonstrate that a quantal response statistical equilibrium approach to the US housing market with the help of the maximum entropy method of modeling is a powerful way of revealing different characteristics of the housing market behavior before, during and after the recent housing market crash in the US. In this line, a maximum entropy approach to quantal response statistical equilibrium model (QRSE) is employed in order to model housing market dynamics in different phases of the most recent housing market cycle using the S&P Case Shiller housing price index for 20 largest- Metropolitan Regions, and Freddie Mac housing price index (FMHPI) for 367 Metropolitan Cities for the US between 2000 and 2015. Estimated model parameters provide an alternative way to understand and explain the behaviors of economic agents, and market dynamics by questioning the traditional economic theory, which takes assumption for the behavior of rational utility maximizing representative agent with self-fulfilled expectations as given.

## 1. Introduction

**Figure 1:**
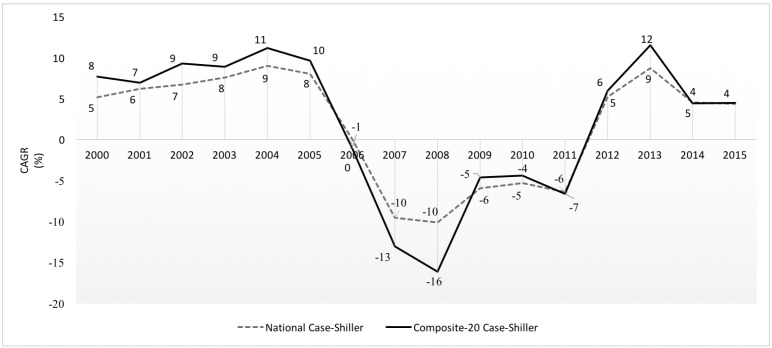
Average (Compound) Annual House Price Growth Rate, CAGR is calculated based on monthly inflation adjusted Case-Shiller National and Composite-20 Indices as: CAGR = ${\left(\frac{\mathrm{Last}\mathrm{Month}\mathrm{House}\mathrm{Price}\mathrm{Index}}{\mathrm{First}\mathrm{Month}\mathrm{House}\mathrm{Price}\mathrm{Index}}\right)}^{\left(\frac{1}{\mathrm{Number}\mathrm{of}\mathrm{Years}}\right)}-1$.

The US housing market experienced one of its key periods of boom-bust cycle between 2000 and 2015, urging researchers to understand and explain the movements of house price changes throughout the cycle. As stated by many authors [1,2] and evident from Figure 1, between 2000 and 2005, seems to enter a positive price feedback loop–in which any house price increase brings about a further rise in house price growth rates, signaling a boom period. Beginning in 2006, the rapid house price growth reverses with slowing appreciation, followed by a catastrophic collapse between 2007 and 2008, in which house price growth rates simultaneously drop nationwide. Decline in house price growth rates alleviates in 2009, but does not experience positive growth again until 2012. The period 2007–2009, therefore, displays a distinct divergence from the periods of 2000–2006 and 2010–2015.

**Figure 2:**
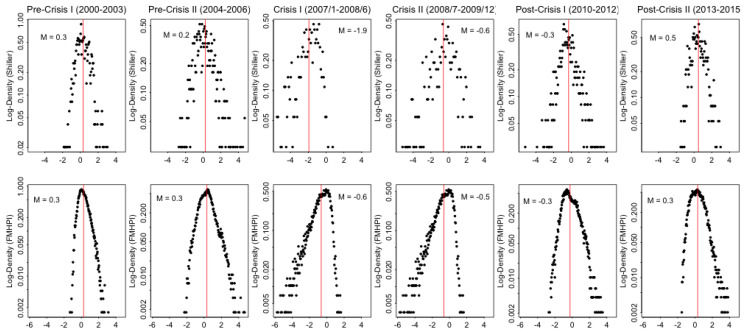
Log Density Plots of Monthly House Price Growth Rates: Calculated as the histogram plots on a log density scale of house price growth rates. The first row shows the histogram plots on a log density scale of monthly house price growth rates based on Case-Shiller Price Index growth rates (%) for each 20 largest metropolitan areas while the second row is based on FMHPI, including monthly price growth rates for the DC region and 367 metropolitan areas in the US. “M” represents the average growth rate for the panel data in each window. Each plot includes all the cross sectional data represented by the specified index, and for the specified time period. Pooled house price growth rate data is then used to calculate log density distributions. More detailed explanation about the data sources will be provided in Section 2.

To capture the behavior of the US housing market for several sub-periods of before, during and after the market collapse, Figure 2 represents the log density distributions of monthly house price growth rates for all available metropolitan areas represented in two different monthly house price indices, Case-Shiller Housing Price Index and Freddie Mac Housing Price Index (FMHPI). Distributions have a striking single peaked shape throughout the cycle indicating a central tendency of the system around an average monthly growth rate. Some distortions in the distribution appear to arise at the end of the boom (2000–2006) and during the crash (2007–2009) by pointing out a change in the characteristics of the market behavior.

Over the past couple decades, several modeling approaches have been put forward to explain large fluctuations in housing markets, such as the one represented in Figure 1 and Figure 2. Search and Matching models of housing markets [3,4], for example, represent temporary house price movements away from the “competitive Walrasian equilibrium” as a result of “exogenous” shocks affecting the demand supply schedules in the market via market frictions, e.g., vacancy rate in the market in which one of the most important problems of modeling interactions among the individuals is reduced to the bargaining process between “homogeneous” sellers and buyers. Simple heterogeneous agent models (HAMs) carry the analysis one step further by introducing two (or three) different types of individuals and their interactions with each other [5,6]. As a result, they are able to reproduce endogenous fluctuations caused by the interactions between heterogeneous individuals (e.g., Chartists vs fundamentalists); however, they use some other simplifications, such as stationary rules to represent the behaviors of individuals and their interactions, and the rules to represent the law of price motions which also raise questions about how realistic the model results are in explaining the real causes behind the market behaviors. Computational Agent Based Models (ABMs) try to solve the problems of HAMs by providing an evolutionary process for the determination of behaviors of heterogeneous agents [7,8,9]. They are able to model a larger scale of more complex interactions between the agents as the main cause of endogenous out-of-equilibrium dynamics of the markets. On the down side, their highly complex and ambitious agenda complicates the estimation process of the model which weakens the robustness of the estimation results. Additionally, since the ABMs are structurally complex, they blur the causal relationships behind the market behaviors.

The Quantal Response Statistical Equilibrium (QRSE) model, introduced by Scharfenaker and Foley [10] employed in this paper, provides a method to explain the observed regularities in highly complex housing markets by taking into account social interactions between a large number of heterogeneous buyers/sellers and their reactions to house price changes. The main objective is to analyze the market dynamics in a simple but realistic way without relying on ad-hoc normative assumptions (For example, models based on traditional economic theory assume complete and transitive set of preferences for “homogeneous” individuals so that they can reduce the problem of providing a micro-foundation for the macroeconomic system to a simple aggregation of all “representative agents’ ” decisions). The model is built upon three main concepts: Statistical equilibrium of targeted economic variable(s) representing the macroeconomic state of the system, the assumption of quantal response behavior of the market participants, and the maximum entropy principle as a method of inference. In this model, the concept of statistical equilibrium from statistical mechanics (pioneered by, Boltzmann [11], Gibbs [12], Maxwell [13] and Jaynes [14]) allows for the relaxation of traditional assumptions of equilibrium. Therefore, deviating from the traditional concept of Walrasian (General) Equilibrium, the QRSE approach focuses on information theoretic *statistical equilibrium*, in which equilibrium takes the form of a distribution representing all possible states of the system (QRSE models introduce a general framework for equilibrium where the conventional market clearing general equilibrium exists just as a special and highly unlikely state of the economic systems). The maximum entropy principle as a method of inference, is the least biased method among all, because it maximizes the uncertainty of the system and gets the least informative state with no additional assumptions other than the existing knowledge of the researcher and observed data. Therefore, it is a useful tool for dealing with the hardship of modeling complex systems, such as housing markets. Finally, by introducing an assumption on the behavior of “purposeful” individuals represented as quantal response logit functions, QRSE models deal with modeling heterogeneity of interacting agents, hence reject the assumption of rational representative agent and replace it with boundedly rational typical agent whose preferences do not have to be well-defined, complete, and transitive. As a result, QRSE models introduce a platform to model social interactions among heterogeneous individuals, uncertainties in individuals’ decision making processes, and endogeneity of the system dynamics without imposing any ad-hoc assumptions.

In this study, observations of the US housing market between 2000 and 2015 from Figure 1 and Figure 2 are used to study the questions of “which forces behind the market dynamics give rise to the boom-bust cycles and the collapse, and how can one capture them?”. To achieve this goal, a Quantal Response Statistical Equilibrium Model is introduced, in which two systemic forces govern equilibrium/out-of equilibrium dynamics of the US housing market. To define these market forces, the model sets a behavioral assumption that typical agents, based on the house price changes in the market, try to maximize the expected returns from their transactions subject to a constraint on the entropy of their mixed strategy over transactions. Moreover, the actions of buying/selling in the housing market create a negative feedback effect on the house price growth rates themselves. Therefore, both the central tendencies in the distribution of house price growth rates and the deviations around these tendencies are the natural results of this process governed by the relationship between the changes in house price growth rates and the actions taken by the individuals in the system.

Based on Figure 2, we argue that periods before and after the market collapse of 2007 indicate a strong statistical equilibrium while the distortion in the equilibrium state starts to rise around late boom period of 2004–2006, and crisis of 2007–2009 signals a strong divergence from the equilibrium. After the crash, the system seems to recover towards a new statistical equilibrium between 2010–2015.

This paper sets out to (i) analyze the observed regularities in the housing market during the most recent housing cycle based on the statistical equilibrium approach, (ii) provide a model to explain the systemic forces behind the behavior of the housing market for the consequent periods of the cycle, and (iii) combine the model results with a solid economic interpretation. Section 2 will present the data sources employed in our analysis, and discuss observed regularities and the divergence from these regularities in the US housing market based on house price growth rates for a large number of metropolitan areas between 2000 and 2015. In Section 3, a maximum entropy method to quantal response statistical equilibrium model by Scherfenaker& Foley (2017) will be applied to the housing market dynamics. Section 4 will focus on the results of the model fit estimations for each sub periods and robustness of parameter estimations. Finally, Section 5 will provide a brief discussion about the results.

## 2. Observed Regularities in the House Price Growth Rates

Sampling and modeling techniques of repeat sales house price indices have been largely criticized in the literature [15]. To set an argument on a sound basis and check the robustness of the results, two different monthly house price index data for several metropolitan areas were used. First one is Freddie Mac House Price monthly index, FMHPI, which is available for DC area and 367 Metropolitan statistical areas (MSAs) throughout the US, and the second one is monthly S&P Case-Shiller Index for 20 largest US metropolitan cities (See Appendix A for more details about each data sets). To capture the regularities in the data, both data sets are deflated using the consumer price index (CPI). Corresponding real house price indices are then, used to calculate monthly real house price growth rates for each region in both data sets. Inflation adjusted monthly house price growth rates, hence, are calculated as $x_{t}=\frac{100(g_{t}-g_{t-1})}{g_{t-1}}$, where $g_{t}$ is the house price index for time *t*.

Growth rates of price indices rather than the levels, are used in this analysis because the rate of change of housing prices play a more significant role than the levels of prices during the decision making process of economic agents because it provides information about the possible returns on the investment, and since we are dealing with cross sectional data, which each region carries different characteristics across time (i.e., peak levels of house prices, and timing to reach the peak differ among different regions), it is more convenient to use house price growth rates as a metric. Also, the rate of change of prices in logarithmic scale is able to capture the exponential or power law types of distributions from the data. It is important to capture extreme values in the data especially during the market crash since house prices do not follow random walk. Instead, they seem to persist in the short run while there exists a mean reversion in the long run [16,17] (Random walk theory pre-supposes the efficient market hypothesis, in which profit maximizing agents are rational, and can reach all available information freely in the market. Therefore, on the average, competition will cause the full effects of new information on intrinsic values of assets to be reflected “instantaneously” in actual prices, see [18]).

### Visualizing Statistical Equilibrium: Patterns of House Price Growth Rates (2000–2015)

As argued by Jaynes [14], Farjoun and Machover [19], Foley [20], and Scharfenaker and Semieniuk [21], the statistical equilibrium framework (from statistical mechanics) to describe macroeconomic systems, is a highly suitable approach, because laws governing macroeconomic variables such as price changes and profit rates carry a statistical character, so that one must employ probabilistic considerations in order to capture such laws. Statistical mechanics was pioneered by Boltzmann [11], Maxwell [13] and Gibbs [12], in order to explain the physical macroscopic properties of matter from the microscopic properties of atoms and molecules which comprise the system as a statistical large population. In developing the kinetic theory of gasses, Boltzmann [11] derived that pressure and temperature of an ideal gas are a function of the average kinetic energy of its particles. Since determination of each micro-state requires specification of the position and momentum of each gas molecule, which is a cumbersome task due to high degree of freedom for the entire system, the theory of statistical mechanics reduces the problem to analysis of macroscopically observable parameters via a statistical analysis of the molecules of which gas is made up. Probabilistic reasoning, hence allows us to reach informative results concerning the equilibrium behavior of the complex systems without relying on strong assumptions about the detailed microscopic behavior of the individual components of the system [14,19].

Based on the same logic, statistical equilibrium in social systems; such as housing markets, also result from the interaction of a large number of economic agents with a certain level of uncertainty in their actions which in turn, collectively have an impact on the outcome variable, such as house price growth rates. Following classical political economy tradition of [22,23], we argue that individual economic agents seeking for a higher return than the economy-wide average from their transactions in a market would generate a “tendential gravitation” of each sub-market’s house price growth rate around that average as an unintentional result of the interactions among many individual buyers and sellers. Therefore, their decisions in the process of competing for higher expected returns determine the change in house prices at any point in time creating an average rate of price change in the market as a whole. This process can be interpreted as the statistical equalization process of house price changes in the system. As a result, statistical regularities (single peaked Laplace-like log frequency distributions) occur due to buying and selling decisions that are conditional on the expected changes in the housing prices for maximizing their returns from their transactions. The process of deciding when and where to sell/buy works as a competitive process, and creates a negative feedback mechanism while payoff maximizing sellers are seeking for the regional price increase to be above their expected fundamental rate of price increase of their houses to sell, and buyers are looking for markets whose regional price increase is below their expected fundamental rate of price increase to buy. Unintentionally, actions of sellers cause a decline in housing price increase in sub-markets, while behavior of buyers causes an increase in housing price change. Concept of statistical equilibrium as a remarkable representation of economic-laws based on classical political economy tradition, hence, can help us to capture the governing forces behind the system’s central tendency and deviations from it. This way we can detect and interpret empirical regularities in the housing market.

As briefly mentioned in introduction, Figure 2 shows the log density distributions of monthly house price growth rates of the panel data for “pre-crisis periods (2000–2003) and (2004–2006)”, “Crisis periods (2007–June 2008) and (July 2008–December 2009)”, and finally “Post-Crisis periods (2010–2012) and (2013–2015)” for two different cross sectional house price index data mentioned above in order to determine statistical equilibrium dynamics in the US housing market. Each plot includes monthly real house price growth rates for each and every metropolitan area represented in the data for the specified periods. This way, one will be able to observe the evolution of the distribution of house price growth rates; regularities during the statistical equilibrium periods, and the divergence from the equilibrium during the crisis, if they exist.

For both data sets, log density distributions of monthly real house price growth rates for the US housing market seem to follow a symmetric Laplace-like single peaked distribution between 2000–2003 and 2010–2015 suggesting a strong statistical equilibrium in the housing market before and after the collapse. Results from FMHPI data emphasizes changes in skewness among the sample periods, while results from the Case-Shiller index data emphasizes the loss of definition of the distribution in the late pre-crisis II and crisis periods. According to results based on FMHPI index, between 2004 and 2006, symmetry in the system starts to deteriorate although the market forces still try to push the system back to the equilibrium. Symmetry in the distribution is not a necessary criterion for the statistical equilibrium. However, it can be interpreted as an indicator of rapid appreciation/depreciation of house prices in some sub markets before/after the crash. When the house market collapses, during 2007–2009 distribution becomes more dispersed for the Case-Shiller House price index signaling a deterioration of market forces whose roles are to push the system to the statistical equilibrium, while FMHPI distributions remains highly skewed to the left implying the increasing house price depreciation in some metropolitan areas (Note: Although each data sets are used for the same purpose in the analysis, some variation between the two is expected because S&P Case-Shiller house price index represents 20-largest metropolitan cities such as Chicago, Las Vegas and New York, while FMHPI consist of DC region and 367 metropolitan areas throughout the US representing a finer grained data. However, results follow similar patterns through time).

Another way of examining the central tendencies for house price growth rates is to look at the average price growth rate movements in the system as a whole. This is because when the system is at or near the statistical equilibrium, in the sense that macroscopic conditions and macroscopic parameters, such as average change in prices, appear to be constant or vary comparatively slowly [19]. Yet, this stable conditions in equilibrium is still dynamic and statistical, allowing microscopic parameters such as house price growth rate in any sub-market to change rapidly. The system as a whole evolves through time so do the macroscopic parameters. When the system is disrupted by a period of crisis, macroscopic parameters in hand can change drastically.

**Figure 3:**
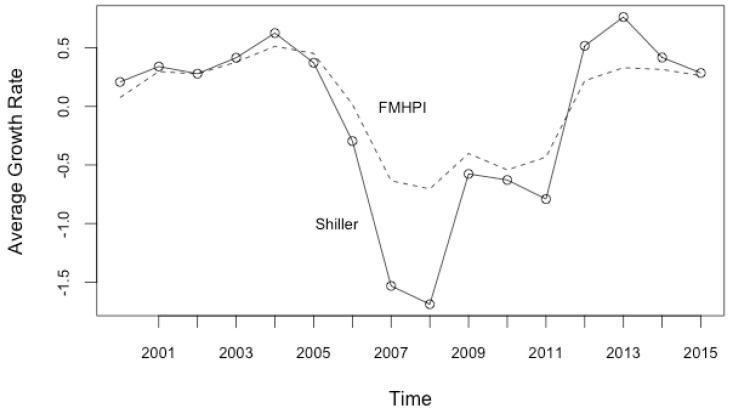
Average Monthly Housing Price Growth Rates (%). Calculated as the average price growth rate of the pooled cross sectional data for each year.

As Figure 3 points out, average monthly price growth rates calculated as the average price growth rate of the pooled cross sectional data for each year for each data sets are quite stable between 2000–2003 around 0.2%–0.34% with a further increase between (2003–2005), while the crisis period shows meaningful changes, in which stability in house price appreciation is replaced by a drastic decline in growth rates around −1.9% for Shiller house price index. The decline in average price growth rate is more moderate in FMHPI, which can also be interpreted as the indicator of severity of crisis experienced by different metropolitan areas. This may be because Case-Shiller price index include 20 largest metropolitan cities which experienced the largest price appreciation during the boom, and the largest depreciation during the crisis, while FMHPI covers DC region and 367 US cities causing finer results on average. This might be signaling one of the stylized facts of US housing market, which is that different metropolitan areas experienced different price appreciation (depreciation) before (after) the crisis. This is why focusing on only the national data would leave out some of the most important characteristics of boom and bust cycles in the housing markets. One can argue that the stability of average monthly house price growth rates during the statistical equilibrium of the pre and post-crisis periods indicates some systemic forces in the housing market, which are dysfunctional during the crisis, and also the purpose of this paper to explain.

For the sake of understanding the forces that push the housing market to the statistical equilibrium, it is crucial to keep in mind that housing market dynamics involve social interactions of a large number of economic agents through socially determined structures in the market, from which the statistical distribution of housing price growth rates is determined. As a result of these interactions, based on individuals’ quantal actions (buy and sell), and their engagement with the resulting outcomes of these actions in a highly “competitive” environment, system generates complex dynamics, which are hard to capture from the data directly. As Scharfenaker and Foley (2017) argues, the difficulty of capturing the dynamics of the economic systems from the data arises due to the dependency of observable variables on unobserved ones, which in turn, causes incomplete information about the problem in hand, raising the importance of the method of estimating the parameters in the model.

In the next section, the maximum entropy-quantal response model of statistical equilibrium, called “QRSE”, introduced by Scharfenaker and Foley [10], will be applied to housing market to overcome incomplete information problem in modeling, and to explain the dynamics/behaviors of the system during the most recent housing market cycle (2000–2015).

## 3. A Quantal Response Statistical Equilibrium Model of the Housing Market

The Quantal Response Statistical Equilibrium (QRSE) model predicts the equilibrium as the most probable state of the system in the form of the statistical equilibrium probability distribution of monthly house price growth rates, f[x], which is derived by maximizing the entropy of the unknown distribution, H[f[x]]=-∑f[x]Log[f[x]] subject to constraints including all information from the observations or theory related issues [14,24] (See Farjoun and Machover [19] Foley [25], dos Santos and Scharfenaker [26], Scharfenaker and Semieniuk [21] and Yang [27] for other applications of the maximum entropy statistical equilibrium approach). It includes two variables, an action variable, *A*, and an outcome variable x:X→R. In this case, the action variable is a binary variable, $A=\{a,\bar{a}\}$ where *a* and $\bar{a}$ represent selling and buying actions respectively, and the outcome variable, *x* represents the monthly real house price growth rate (%) (In real applications, measurement based on *x* variable will be coarse-grained in finite number of bins). The heart of the model lies on the interaction between the action and outcome variable.

The model, which takes the interactions among buyers and sellers into account, is a joint distribution of actions A={sell,buy} and house price growth rates, *x* which will be written as f[A,x] with the marginal and conditional frequencies f[x],f[A],f[A|x],f[x|A]

f[x]=∑f[A,x];f[A]=∫f[A,x]dx

$$f\left[A|x\right]=\frac{f[A,x]}{f[x]}\;\mathrm{if}\;f\left[x\right]>0,f\left[x|A\right]=\frac{f[A,x]}{f[A]}\;\mathrm{if}\;f\left[A\right]>0.$$

In this set up, conditional distributions represent the causal relationships and hence, include the explanatory power for the model to overcome the problem of incomplete information. Quantal response of individuals’ actions assumes that individuals make their decisions based on the outcome variable, in this case, house price growth rates. Therefore, not only do individuals’ actions (selling/buying) affect the house price changes (*x*), but the change in house prices also affects individuals’ selling/buying decisions.

The interdependence between selling/buying actions of individuals and the change in house prices are therefore, represented by the conditional action distributions:

(1) $$f\left[A|x\right]=\{f\left[a|x\right]=f\left[sell|x\right],f\left[\bar{a}|x\right]=f\left[buy|x\right]\}.$$

The causal relation that links the buying-selling behavior of individuals to the house price growth rate, *x*, symmetrically determines the conditional outcome frequencies;

(2) $$f\left[x|A\right]=\{f\left[x|a\right]=f\left[x|sell\right],f\left[x|\bar{a}\right]=f\left[x|buy\right]\}.$$

Overall, conditional frequency distributions Equations (1) and (2) allow us pursuing a theory of interaction through the inter-dependency of actions and the outcome variable in the case of housing market representation. Therefore, statistical equilibrium in the housing market model is a joint distribution, which determines two conditional distributions, f[A|x] and f[x|A].

The problem of modeling the housing market occurs because we end up having house price changes as outcome variable, which is determined by individual actions in the housing markets. In general, if one knows one of the conditional distributions via observations or theoretical considerations, and also the corresponding marginal distributions from observations, such as f[A|x]andf[x], then they would be enough to determine the joint distribution f[A,x]=f[A|x]f[x] from which one can derive the other marginal and conditional distributions. In this case, observed data would provide full information to estimate the statistical equilibrium. Yet, most of the time this is not the case. For example, in our case, we only have the marginal distribution of house price changes f[x], and need to learn about the conditional distributions f[A|x] and f[x|A]. Buying-selling actions of individuals could be easily observable for some data sets, but for our data sets it is not the case. Individual actions of house buyers and sellers cannot be observed simultaneously apart from house price growth rate data. This means, when we are observing house price changes, we cannot measure the intensity of buying and selling actions of individual agents in the housing market from the same data directly. Here, we want to estimate the joint distribution in order to infer the causal links, but we only have a partial information about the joint distribution, such as observations on house price growth rates that determine the marginal distribution of housing price changes f[x].

This type of a quantal response social interaction problem with only a partial information about the joint distribution of quantal actions and the outcome variable that is determined by these actions, such as an observed marginal distribution, necessitates using a theory that offers a prior over one of the conditional distribution using Shannon’s information theory [28], and Jaynes’ (1957) principle of the maximum entropy inference. This means model constraints in the entropy maximization problem play the key role.

### 3.1. Model Constraints

As emphasized by [24,29], it is important to choose the right constraints either using the observable data or theoretical frameworks, when employing the maximum entropy program to generate the most likely statistical equilibrium distribution of the system. Here, there are two important constraints, which are needed to represent the housing market dynamics. The first constraint represents the behavior of the individuals in the housing market based on the quantal response, and the second one is based on the impact of individuals’ actions on the house price changes.

#### 3.1.1. Constraint 1: The Impact of House Price Changes on the Actions of Economic Agents (Quantal Response Behavior of Individuals)

Traditional economic theory assumes that decision makers have complete and transitive preferences over outcomes represented by a utility function [30,31,32]. According to this approach, when utility maximizing rational individuals face with a decision to buy/sell a house in a particular market based on their payoff functions (i.e., the difference between the rate of increase in prices in the region and their estimate of the fundamental rate of price increase of the house), they would almost never sell (buy) the house when prices in the region rise slower (faster) than their expected fundamental rate of price increase of the house, and would wait until their expectations about house price increase coincide with actual price increase in the market (See Foley [33] for more methodological and theoretical details).

Yet, as it has been shown both experimentally and empirically, this approach does not represent the reality [34,35]. To provide a more realistic representation of individual behavior, the first constraint of our model assumes that individuals follow a binary quantal response function when they decide to sell/buy in the market. Therefore, we argue that when the regional price increase is well below sellers’ estimate of fundamental rate of price increase of the house they would almost never sell. However, when the regional price increase gets closer to their expected rate of fundamental increase of the house, they sell more frequently depending on the regional price increase; even though their expectations about the price increase of the house do not perfectly coincide with the actual price increase in the region. Opposite is true for the buyers. When a buyer’s estimate of fundamental rate of price increase for a house is well above the regional price increase (regional price appreciation rate is smaller than what she/he expected), they would almost always buy. However, when the actual regional increase gets closer to their estimates, they would buy less frequently.

**Figure 4:**
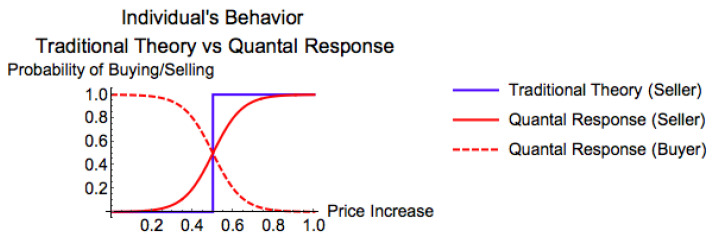
Individual’s behavior: Traditional Theory vs. Quantal Response.

Figure 4 demonstrates the difference between these two approaches in explaining the behavior of a seller/buyer as explained above. According to traditional theory, a rational individual would follow a step function while a typical individual in our model follows a logistic S-curve.

Modeling quantal response of market participants in the maximum entropy framework assumes that the agent may respond to a payoff function u[A,x]. In the case of housing market, it represents the payoff to the typical individuals who sell/buy houses in the market. If the agent chooses a mixed strategy f[A|x]:AxX→(0,1) over actions $\left\{a,\bar{a}\right\}=\left\{sell,buy\right\}\in A$ to maximize the expected payoff subject to a constraint on a lower bound on the informational entropy of the mixed strategy, $\sum_{A}f\left[A,x\right]u\left[A,x\right]$ as:

$$\begin{array}{cc} \underset{x}{\mathrm{Max}} & f[A,x]u[A,x]\\ \mathrm{subject to} & \sum_{A}f[A,x]=1\\ & H\left[f_{A,x}\right]=-\sum f\left[A,x\right]Log[f\left[A,x\right]\geq H_{min} \end{array}$$

the resulting function turns out to be the quantal response (Gibbs) function $f\left[A|x\right]\propto e^{u[A,x]/T}$. When the parameter T>0, recovered link functions will assign probabilities to different responses which are represented by S-shaped logit quantal functions for selling and buying actions.

Scharfenaker and Foley [10] presents the difference between the payoffs of selling and buying as a linear function by introducing a shift parameter μ, defined as $u\left(a|x\right)-u\left(\bar{a}|x\right)=x-\mu$. Here, *x* is the regional house price change while μ represents the expected fundamental rate of price increase for the house which the agent is willing to to sell/buy. This relation tells us that higher house price growth rate increases the payoff of selling action while payoff of buying diminishes, thus the gap between the payoffs increases. Moreover, if the gap between the two is zero individuals become indifferent between buying and selling. In this case, μ determines the indifference point of the house price growth rate in which the probability of selling is 50%. It provides useful information about the willingness of market participant’s to sell at a certain house price growth rate, which will be discussed in more detail in following sections.

With this new specification, the impact of house price changes on the actions of selling and buying will be modeled as:

(3) $$\begin{array}{c} \begin{array}{c} f\left[sell|x\right]=\frac{1}{1+e^{-\frac{\left(x-\mu \right)}{T}}},\\ f\left[buy|x\right]=\frac{e^{-\frac{\left(x-\mu \right)}{T}}}{1+e^{-\frac{\left(x-\mu \right)}{T}}}. \end{array} \end{array}$$

As a result, introducing an informational entropy constraint into the maximization problem allows one to introduce the bounded rationality framework explaining the individual’s decision making process. Therefore, we are able to take into account individual’s uncertainty of her/his actions which could be resulted from difficulty in reaching the perfect information, or in assessing market signals etc. during her/his decision making process (See Sims [36]). This way, we discard the the assumption of “representative rational agent”—with complete and transitive preferences over price increases—which is the backbone of the traditional theory.

#### 3.1.2. Constraint 2: The Impact of Actions on the House Price Growth Rates

The second constraint that will help us to model the impact of selling/buying actions on house price changes in the market is the conditional outcome constraint. As mentioned before, the act of selling houses itself tends to lower the house price growth rate in the sub-market relative to what it would have been if selling behavior had no downward pressure on the price change in the market. The effectiveness of buying and selling process can be represented in a maximum entropy formalism by a constraint on the difference between the weighted expected house price growth rates conditional on selling and buying actions of the individuals which can be written as:

(4) $$\begin{array}{c} f[sell]E[x|sell]-f[buy]E[x|buy]\leq \delta \\ \int (f[sell,x]-f[buy,x])xdx\leq \delta \end{array}$$

where δ represents the “effectiveness” of the competition in the market. When δ gets larger, the effect of selling and buying actions on the house price growth rate worsens indicating a deterioration in negative feedback mechanism from actions to house price change.

Based on these two constraints, it is argued that individuals make their decisions to sell or buy a house by comparing the house price increase in a particular region with their “expected fundamental rate of price (value) increase of their houses”. To illustrate, in a housing market, sellers tend to sell more often if house price increase in the region is higher than her expected fundamental rate of price increase of the house expecting lower returns in the future. On the other hand, buyers tend to buy more often if the regional price increase is lower than their expected fundamental rate of price increase of a house expecting more appreciation in price increase for the house in the future. The collective result of the actions, in turn, is strong enough to impact the house price growth rate in the market (It is argued that each individual’s action creates a “small” impact on regional price changes). This means, when sellers sell their properties in a particular market, their actions set forces for a negative feedback mechanism on the house price change, and house price growth rate declines in the market. The opposite happens when the buyers buy houses so that upward price changes tend to increase. As a result of this statistical process, actual transactions among typical agents give rise to a statistical equilibrium distribution of house price growth rates around an average which the system tends to evolve towards. The regularities in the system, therefore is represented as a distribution of house price changes.

### 3.2. Solution to the Maximum Entropy Program of the QRSE Model for the Housing Market

Given the observed frequency data for monthly house price growth rates $\bar{f}\left[x\right]$, the theory of dependency of buying/selling actions on house price growth rates, and dependency of house price growth rates on actions, we aim to infer a quantitative model of housing market behavior in the form of a joint probability distribution f[A,x] over the action, *A*, and observed house price growth rates by using the maximum entropy method.

As the result of maximizing entropy of the the joint distribution subject to three constraints —the constraint on the difference between the weighted conditional expected outcomes, Equation (4), a constraint on the mean value of the outcome, ∫f[x]xdx=ξ, and the assumption that the conditional action is a logit function, Equation (3) with parameters μ and *T* – the maximum entropy (recovered) marginal distribution $\hat{f}\left[x\right]$ is inferred as the most probable distribution representing the statistical equilibrium in the housing market as (See Appendix B for the derivation of the maximum entropy program):

(5) $$\hat{f}\left[x\right]=\frac{e^{H_{\mu ,T}\left[x\right]}e^{-\gamma x}e^{\beta Tanh[\frac{x-\mu }{2T}]x}}{\int e^{H_{\mu ,T}\left[x\right]}e^{-\gamma x}e^{\beta Tanh[\frac{x-\mu }{2T}]x}dx}.$$

$\hat{f}\left[x\right]$ is predicted as a Kernel to the maximum entropy program, and together with T,μ,β and γ provides us with a multinomial model of the house price growth rates. The hyperbolic Tangent function, (Tanh), which also is a symmetric function, represents the kurtosis of the system, and determines how spread out the marginal distribution can get.

*T* is called “responsiveness” parameter, and gives information about how the change in house prices affect the behavior of the individuals. Therefore, it is useful to understand how strong is the relationship between the outcome and the action, i.e., how much the change in house price growth rate affects the decisions of individuals’ action of selling houses. It provides useful information about the fluctuations and uncertainties in agents’ decision making process. The impact parameter β, which is the Lagrange multiplier for δ, measures the impact of intensity of actions on the sub-regional outcomes i.e., how the actions of agents (selling/buying) affect the house price growth rate in a particular region. Another important parameter, μ represents the expectations of individuals about the fundamental rate of price increase for a house, which can also be interpreted as perceptional behavior point in which the agents are active buyers and/or sellers in the sub-regions. Therefore, equilibrium behavior of the system could be measured as a combined result of parameters T,μ and β. Parameter γ represents the shadow price for the mean constraint, which reveals information about the skewness of the marginal distribution. It also helps us to understand the relationship between expected fundamental rate of price increase for the house, μ, and actual statistical average price increase in the data, ξ. γ becomes zero if μ=ξ, while γ<0 represents positive skewness in the marginal distribution (See Appendix C, for the posterior probability estimation of the parameters, which provides the credible intervals for the estimated parameters).

## 4. Model Inference and Results

**Figure 5:**
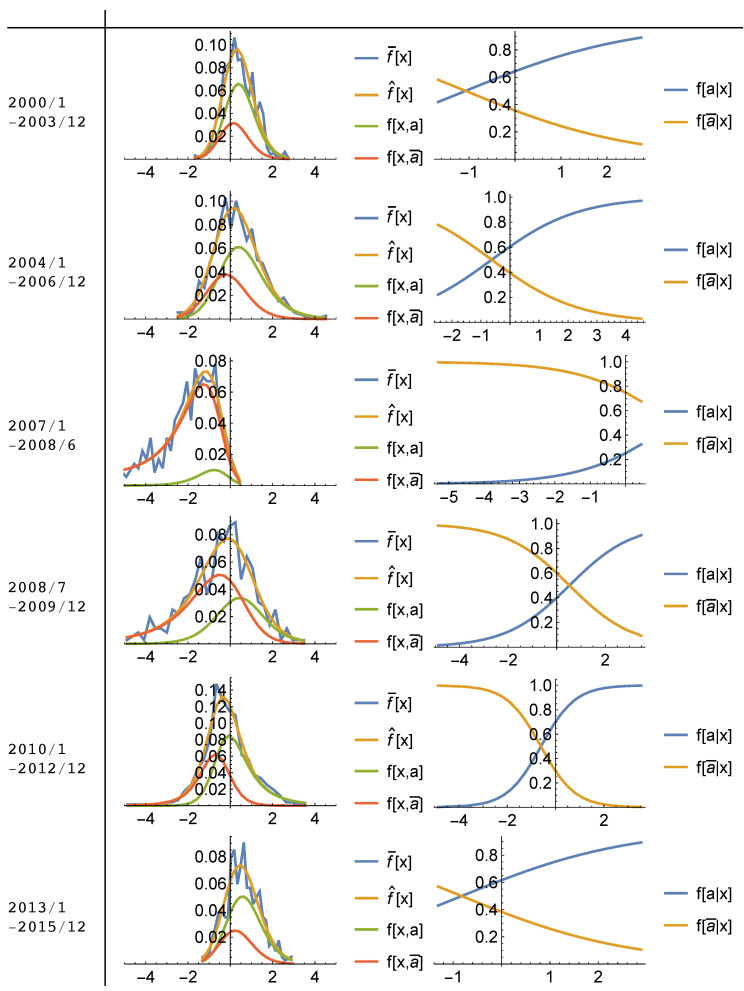
Model Fit Plots (2000–2015) (Based on Inflation Adjusted S&P Case-Shiller Index Monthly House Price Growth Rate for each MSA represented in S&P Case-Shiller Index). Observed marginal frequencies; $\bar{f}\left[x\right]$, fitted (inferred) marginal frequencies; $\hat{f}\left[x\right]$, maximum entropy conditional outcome frequencies; $f\left[x,a\right],f\left[x,\bar{a}\right]$ (**left**), estimated conditional action frequencies; $f\left[a|x\right],f\left[\bar{a}|x\right]$ (**right**), in which *a* represents selling action while $\bar{a}$ represents buying action in the market.

**Figure 6:**
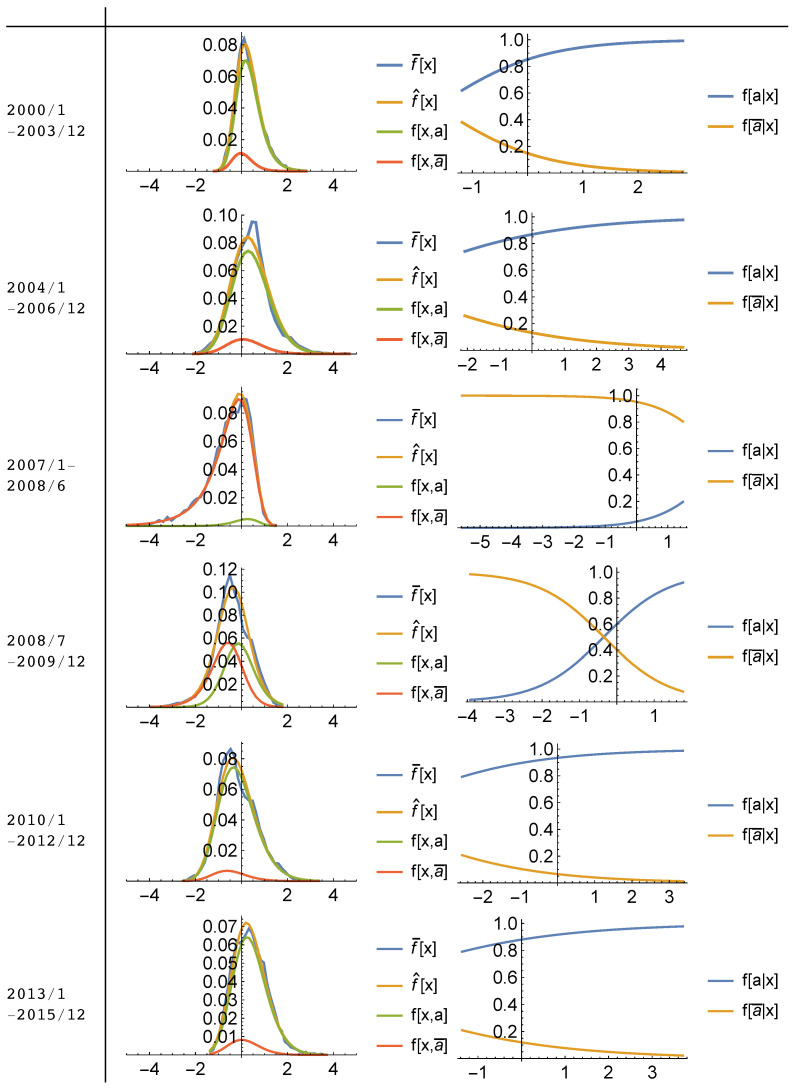
Model Fit Plots (2000-2015) (Based on Inflation Adjusted FMHPI Monthly House Price Growth Rate for each MSA presented in FMHPI index). Observed marginal frequencies; $\bar{f}\left[x\right]$, fitted (inferred) marginal frequencies; $\hat{f}\left[x\right]$, maximum entropy conditional outcome frequencies; $f\left[x,a\right],f\left[x,\bar{a}\right]$ (**left**), estimated conditional action frequencies; $f\left[a|x\right],f\left[\bar{a}|x\right]$ (**right**), in which *a* represents selling action while $\bar{a}$ represents buying action in the market.

Figure 5 and Figure 6 visualize the solution to the maximum entropy program subject to constraints from Appendix B. Parameters are estimated for subsequent time periods; 2000–2003, 2004–2006, January 2007–June 2008, July 2008–December 2009, 2010–2012 and 2013–2015 using the panel data of monthly house price growth rates for the metropolitan areas represented in the data sets. Sub-periods are chosen based on distinctive differences in house price growth rate distributions which are represented in Figure 2 by taking information about historical house price movements in the US housing market into account. Then, for each sub-period, monthly house price growth rates of the entire MSAs represented by the data are pooled together for the estimation. Here, the objective is to capture the evolution of housing market behavior both qualitatively and quantitatively throughout the cycle.

Figures represent the observed marginal frequencies; $\bar{f}\left[x\right]$, the fitted (inferred) marginal frequencies; $\hat{f}\left[x\right]$, the maximum entropy conditional outcome frequencies; $f\left[x,a\right],f\left[x,\bar{a}\right]$ (left), and the estimated conditional action frequencies; $f\left[a|x\right],f\left[\bar{a}|x\right]$ (right), in which *a* represents selling action while $\bar{a}$ represents buying action in the market.

Table 1 presents the values of the estimated parameters and related variables obtained from the model fit results.

Model fit results, show that the observed distributions of house price growth rates can be explained as the statistical equilibrium (or dis-equilibrium for some periods) produced by the interactions of buyers and sellers in the sub-housing markets according to a logit quantal response and a given effectiveness of buying/selling in affecting the house price growth rates in these regions.

As can be followed from the Appendix C in more detail, model parameters T,μ,β and γ, are estimated jointly by minimizing the Kullback-Leibler divergence between the observed marginal frequencies, $\bar{f}\left[x\right]$, and inferred marginal frequencies $\hat{f}\left[x\right]$, where the inferred frequencies $\hat{f}\left[x;T,\mu ,\beta ,\gamma \right]$ is obtained from Equation (5). Results from this process indicate that given the estimated parameters, T,μ,β,γ, the QRSE model generates almost a perfect fit for the observed marginal frequencies in both data sets. Calculated information distinguishability (ID) measures, which show the closeness of the model fit to the observed frequencies, indicates that fitted distributions of marginal frequencies, $\hat{f}\left[x\right]$ capture approximately 96 to 98% of the information content of the observed frequencies for each sub-period.

### 4.1. Interpretation of Estimated Model Parameters

**Figure 7:**
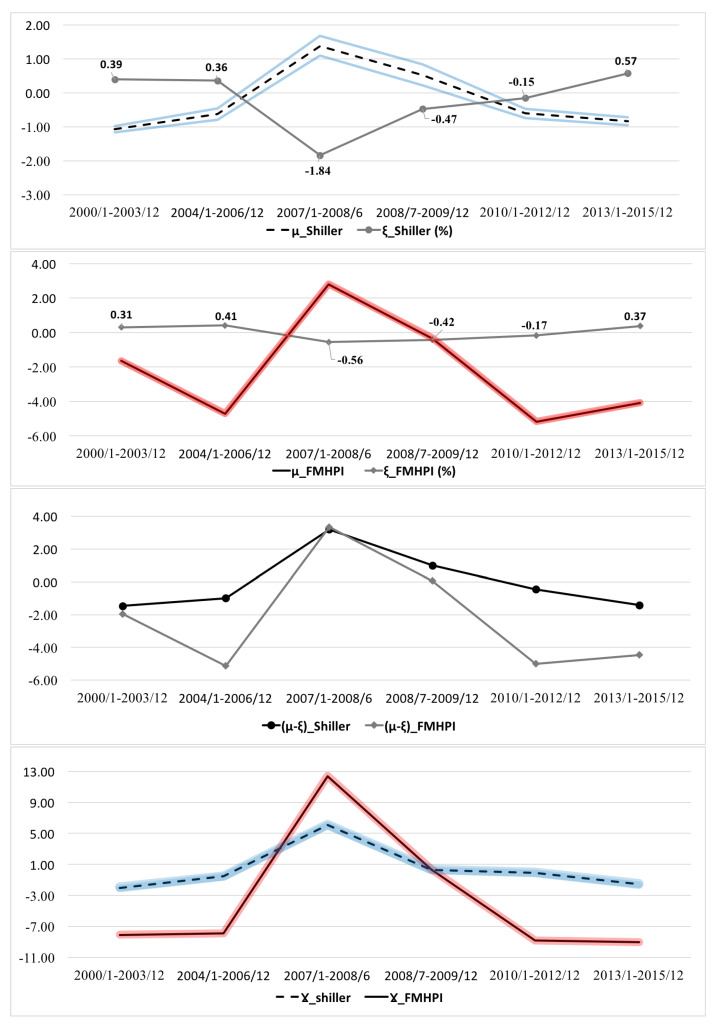
Estimated Parameters with 95% Credible Intervals (HPD): μ, actual average house price growth rate ξ, (μ-ξ) and γ. Red and Blue lines represent the 95% credible intervals for estimated parameters.

**Figure 8:**
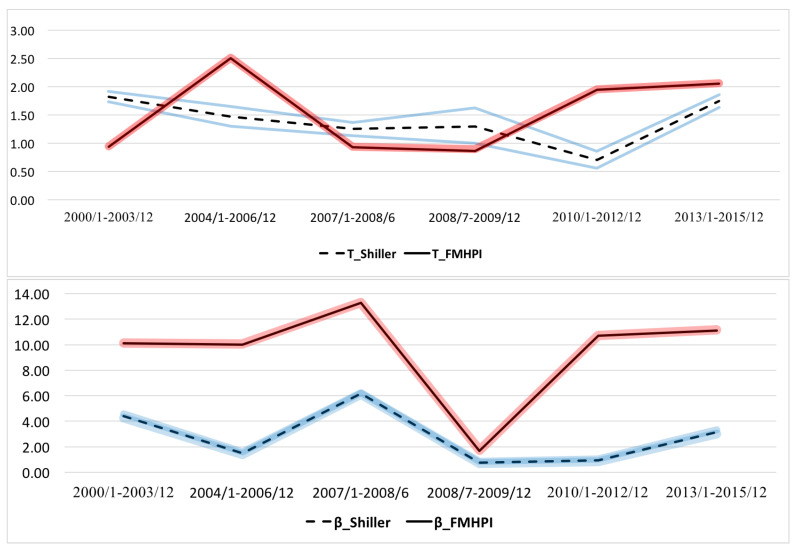
Estimated Parameters with 95% Credible Intervals (HPD): *T* and β. Red and Blue lines represent the 95% credible intervals for estimated parameters.

Using the model fit results, Figure 7 and Figure 8 present the time series of estimated parameters, T,μ,β,γ, with highest posterior density regions (HPD) (See Appendix C).

#### 4.1.1. Interpreting μ, γ and Their Relationship with Actual Average House Price Growth Rate ξ through the Cycle

Parameter μ—expected rate of fundamental price increase of a house—provides useful information about the expectations of economic agents. If μ≠ξ, it means that expectations of individuals about the fundamental house price increase do not coincide with the actual average price increase of the whole market signaling “unfulfilled" expectations of the individuals [33].

As Figure 7 shows, results from the model fit for both data sets indicate that individuals’ expectations about the price increase stay below the actual average house price increase (μ<ξ) before and after the crisis for both data sets. Moreover, as can be followed from the Table 1, marginal probability of selling, marg(*a*) seems to be higher than the probability of buying, marg($\bar{a}$) in the market pointing out that the market participants were more willing to sell before and after the crash. This proves that higher actual (average) price increase than what market participants expected encouraged the sellers (buyers) to supply (demand) their houses more (less) frequently signaling the characteristics of a boom period. This is because the opportunity cost of keeping an empty house for a seller during the boom periods is higher than it is during the bust since there is a chance that house values might decline in the next period [37]; hence, the payoff of selling becomes higher during the boom, while it is the opposite for the bust and crash periods. During the crisis, μ exceeds ξ signaling a significant deterioration in individuals’ expectations and willingness to sell which shows itself as a low probability of selling action because the price changes dropped significantly. After the crisis, their perceptions about the market seem to return to pre-crisis periods where μ<ξ. It is important to emphasize that expectations of economic agents never perfectly coincided with the actual average price increase—μ was never equal to ξ throughout the cycle. Another way of presenting the individuals’ expectations and willingness of selling/buying can be followed by the measure, (μ-ξ), from Figure 8. Increasing gap between individuals’ expectations and actual average price increase becomes more obvious during the collapse. By reaching its peak during the crash, (μ-ξ) with a low probability of selling action renders how overcautious the market participants become when the system enters to crisis.

In addition to the relationship between μ and ξ, another important piece of information that μ provides us with is that housing market was more inclined to be “frothy” and speculative before and after the crash meaning that the market was more vulnerable to create a housing market bubble. This can be observed from the expectation parameter μ being negative for pre and post-crisis periods for both data sets (except during the crash it becomes positive).

The estimation of γ parameter informs us about the skewness of the marginal distributions, which in turn, helps us to interpret the relationship between μ and the actual mean ξ. When μ is equal to ξ, γ=0 indicates perfect symmetry in the marginal distribution, $\bar{f}\left[x\right]$. On the other hand, μ<ξ implies positive skewness, meaning that some of the regions are experiencing house price increases well above the market average. This might mean that the market is actually in the boom cycle and building upon the housing bubble. As can be seen from Figure 8, γ was negative before and after the crash demonstrating a positive skewness in the marginal distributions, which also coincides with the boom periods in the housing markets while house price growth rates were increasing. During the crisis, γ shifts from negative to positive values indicating an increase in skewness to the left caused by negative price growth rates in most of the regions due to the market crash. Based on these results, one can argue that estimated γ parameter is a useful indicator for differentiating between boom and bust periods in the market, and the divergence between the expectations and the real outcome.

Overall, after the early pre-crisis sub-period (2000–2003), sellers start to become more cautious about their actions which can be observed from the relationship between μ, ξ and γ. Although marginal probability of selling was higher than the buying action during the periods before the crash, it starts to decline after 2003, and buying becomes more attractive than before, and the system becomes less positively skewed to the right before the crash because regional prices started to depreciate around late 2006. Periods of crisis, in which sharp declines in the house price growth rates occurred in most of the regions, could be represented as vanishing willingness to sell in the market (increasing μ-ξ), and increasing negative skewness of marginal distribution of the house price growth rates (positive values of γ).

After the crash, both μ and γ decline and get closer to their pre-crisis levels emphasizing the similarities in the behavior of the system between pre-crisis and post-crisis periods.

#### 4.1.2. Interpreting Negative Feedback Mechanism, β, through the Cycle

As mentioned before, β is the Lagrangian multiplier for the impact constraint, δ that limits the impact of selling on the change of house price growth rates in sub-regions. Therefore, it represents the impact of actions on the house price changes providing information about how effective is the market. The lower the β is, the lower the market effectiveness. This, in turn, means that the negative feedback mechanism from actions on house price changes, was deteriorated. For example, in a well functioning market, an increase in house price growth rate in a particular region would attract sellers to sell. Once the actions are taken, if the market has an highly effective negative feedback mechanism, it would cause a decline in house price growth rate of this particular region. On the other hand, if the mechanism does not work properly, house price growth rate in the region would keep increasing.

Time series of estimated β for both data sets from Figure 7 show that impact of selling/buying actions on forcing house price growth rates in the regions towards the average growth rate of the market was much weaker between 2004–2006 than 2000–2003 indicating a deterioration in the negative feedback mechanism right before the crash.This behavior of the market creates a plausible environment for unsustainable housing price bubble before the crash.

During the first half of the crisis, β increases to keep the system in equilibrium but since the willingness of selling action drops dramatically (μ>ξ) which also shows itself as a drastic drop in probability of selling (low supply of houses), even a strong negative feedback mechanism (higher β) is not able to prevent market from collapsing. As a result, after the first half of the crisis, β reaches its lowest level indicating a dysfunctional negative feedback from the actions. To illustrate, if deterioration in the negative feedback mechanism did not occur between 2004–2006, strong willingness of selling action of individuals (higher supply than demand), represented by the relationship between μ and ξ, would cause house price growth rates to decline preventing prices from growing further. Since this is not the case between 2004 and 2006, price growth rates keep appreciating. When the appreciation reaches to unsustainable levels, market collapses in 2007 leading to further decline in the effectiveness of negative feedback mechanism between July 2008 and December 2009. After the collapse, β starts to rise again signaling the recovery of the market forces, which brings the system back to statistical equilibrium.

Overall, the trends in β and μ provide a strong evidence that the cause of the bubble cannot be explained only by a simple supply and demand relation (e.g., the larger demand for the housing than the supply of the housing in the market) because the intrinsic mechanism behind the boom and the housing market bubble was the relationship between the actions and the outcome price changes. Traditional explanations for the housing market behavior such as a positive feedback mechanism on house price appreciation from the buyers, or speculations in the market [2,38] could only explain some part of housing bubble creation. If the negative feedback mechanism between the actions and house price changes worked effectively during the boom, supply of housing by sellers would be able to prevent the bubble from happening, and the system would not be able to diverge from the strong statistical equilibrium of the early 2000’s. Wrong (unfulfilled) expectations seem to feed into both increasing prices and reckless actions (e.g., buyers ready to buy at a higher rate than normal times) without disrupting the bubble creation.

#### 4.1.3. Interpreting Responsiveness Parameter, *T* through the Cycle

As briefly mentioned before, logit parameter from the quantal response behavior of individuals, *T*, determines the endogenous fluctuations of the house price growth rates as an indicator of the responsiveness of the economic agents’ actions to the house price changes. The higher the *T* is the flatter the conditional action frequencies f[A|x]—flatter S-curves—become implying a weaker dependence of selling/buying actions on the house price changes. As a result, fluctuations and individuals’ uncertainty of house price change become large. When *T* gets closer to zero, the dependence of actions on house price changes becomes stronger. This means even a small change in house price growth rate results in an instantaneous response of individuals on choosing their actions.

Responsiveness parameter, *T*, thus provides us with two quantitative measures to interpret the behavior of the individuals in a certain time period. First, it measures how responsive the action frequency of economic agents (buying/ selling) to changes in house price growth rates at the average house price growth rate, because derivative of the conditional action function at x=μ is $\frac{1}{4T}$. To illustrate T=1.82 for the early pre-crisis period (January 2000–December 2003) based on Case-Shiller index, indicates that a 1% increase in the house price growth rate near the average growth rate would raise the frequency of selling action by 0.13%. Based on FMHPI index results, T=0.94 for the same period indicates that the same increase in house price growth rate near the average would raise the frequency of selling action by 0.26%. The difference between the two results tells us that any increase in house price growth rate would be more effective to attract sellers to take action in the market with a smaller *T*, who are more responsive to the changes in house price growth rates.

Second, *T* defines the region where the action is sensitive to house price changes: the value of *x* for which the conditional action frequency is *p*, with 0<p<1 is x[p,T]=TLog[p/(1-p)]. For example, based on Case-Shiller Index data, T=1.82 implies that the region of house price growth rate deviations where the frequency of selling lies between 0.05 and 0.95 is {-5.36%,5.36%} while it is {-2.76%,2.76%} based on FMHPI data for the period of 2000–December 2003. Accordingly, this tells us about how tight the quantal response link functions are (See Figure 5 and Figure 6 for the quantal response link function plots, which represent conditional action distributions f[a|x] and $f[\bar{a}|x]$).

The time series of *T*, estimated from the Case-Shiller Index data in Figure 7 demonstrates a stable pattern with a smooth decline between 2000 and 2009 from T=1.82 to T=1.29. In the early post-crisis period (2010–2012), it drops sharply to its minimum level, T=0.7, and starts to increase dramatically after 2012 nearly reaching to its early pre-crisis period level, T=1.74 in the end of the cycle.

From the FMHPI index, *T* seems to follow a more volatile pattern as compared to results from the Case-Shiller Index. Starting from, T=0.94, in early pre-crisis period, it reaches its maximum level of T=2.5, in late pre-crisis period (2004–2006). A sharp decline in *T* occurs during the crisis (2007–2009) reaching approximately the same level as in the case of Case-Shiller Index data, T=0.86. After the crash, it starts increasing steadily above its late pre-crisis period level of T=2.05 towards the end of the cycle between 2013 and 2015.

Patterns in the behavior of the temperature parameter, *T*, based on Case-Shiller Index data, which represent the largest metropolitan cities in the US, imply that typical sellers/buyers were less responsive to the changes in the house price growth rates at the beginning of the cycle, but they started to eliminate the uncertainties about the house price changes, that in turn, strengthened the dependency between actions and house price changes. In general, one would expect increasing uncertainties of individuals’ actions of buying and selling to changes in house prices when the market gets closer to collapse. Yet, here, individuals seem to build stronger ties to the house price movements before the crisis, which might be due to increasing optimism about the future of the housing market, such that every increase in growth rate of house prices encouraged house sellers to be more responsive to house price increases in the market.

On the other hand, in case of FMHPI index data, which represents a larger number of metropolitan regions, *T* follows a sharp increase during the late pre-crisis period (2004–2006) signaling the worsening responsiveness of individuals’ actions to house price changes. When the crash occurs responsiveness to the market signals seems to be regained but starts to deteriorate again right after the crash.

## 5. Discussion

The relationship between individuals’ expected rate of fundamental price increase of the houses (μ) from the model estimations, which represents the expectations of individuals, and the actual average price increase in the market (ξ) raises important questions about how models based on traditional theory might be misleading. To illustrate, taking the housing market case as an example, the traditional approach [30,31,32] would assume that sellers’ expectations about the price increase of a house would be equal to actual average price increase in the market (μ=ξ). This is because individuals would have “correct (fulfilled)” expectations about the price changes. Moreover, if they had wrong expectations, the market would clear immediately since the individuals with wrong expectations would either be dropped out of the market, or would correct their expectations immediately as they process all available information related to prices. As a result, the differences in price changes in different sub-regions would cancel each other out, and the market would reach to the equilibrium instantaneously such that we would end up having a symmetric frequency distribution of house price changes for the whole market. Additionally, the process of market clearing would not impose any cost on market participants.

Even though, this scenario approximates the time periods where the market wanders around a statistical equilibrium (e.g., 2000–2003 and 2013–2015), it cannot explain the observed positive skewness in the distribution between 2004–2006, and negative skewness during the first half of the crisis (2007–June 2008) in the Figure 5 and Figure 6. As opposed to statistical equilibrium periods, they indicate significant divergence from the equilibrium signaling “unfulfilled (wrong)” expectations of individuals.

In contrast with the traditional theory, this study shows that individuals expectations about fundamental rate of price increases never coincided with the actual average price change of the market (μ≠ξ). Instead, when the expectations about the fundamental rate of price increase was lower than the actual price increase (μ<ξ), sellers enjoyed higher price appreciation than expected while buyers might have had to buy at a “too high rate” believing that prices would have continued appreciating in the future. This market behavior seems to result itself in a boom period which in turn, explains the positive skewness in house price changes before the crash. Since price increase kept appreciating during the boom, positive skewness could be interpreted as a “market punishment for the buyers” since they kept buying at an higher price increase than the normal times due to their optimistic expectations. During the crash, on the other hand, market dynamics took the opposite direction in which individuals’ expectations about the fundamental rate of price increase reached to a higher level than the actual average rate of price increase (μ>>ξ) so that sellers’ willingness to sell declined as they expected higher rate of price increase for their houses than the market average. As a result, a sharp, further decline occurred in the observed actual average price increase (ξ) well below what individuals expected. Even though probability of buying (demand of housing) was much higher than the previous periods, market was frozen due to lack of housing supply (very low probability of selling). The period of crash with a declining house prices in most of the regions shows itself as a negatively skewed frequency distribution of house price changes in Figure 5 and Figure 6. Since the values of houses kept depreciating with declining prices, the negative skewness could be interpreted as the “market punishment for the sellers [33]” during the crisis.

Based on these results, one can argue that housing market does not clear immediately, and the process of getting out of disequilibrium dynamics creates certain costs on market participants.

## 6. Conclusions

In this paper, it has been shown that employing the maximum entropy method with plausible theoretical frameworks as the constraints, we are able to overcome the incomplete information problem resulted from unavailability of data for individuals’ actions. As a result, we were able to fully determine the statistical model explained as an equilibrium joint distribution of observed house price growth rate data and unobservable buy/sell actions of individuals given the predicted model parameters, T,μ,β and γ. The recovered joint distribution of house price growth rates, *x* and individual actions $\left\{a,\bar{a}\right\}$, which implies the marginal distribution of house price growth rates, $\hat{f}\left[x\right]$, was then compared to the observed distribution of *x*, $\bar{f}\left[x\right]$ using the Kullback-Leibler (KL) divergence. Since KL Divergence is a good approximation to log posterior probability for the multinomial model for any parameters, we were able to estimate the conditional posterior distributions of each model parameter holding others at their maximum posterior estimates.

Estimations from the both data sets used in the analysis (S&P Case Shiller Index and FMHPI index) follow strikingly similar patterns throughout the cycle proving the strength of the QRSE model’s explanatory power of defining the behavior and dynamics of social systems.

Predicted parameters of the model T,μ,β and γ reveal significant characteristics of the US housing market dynamics for pre-crisis, crisis and post-crisis periods during the most recent housing market cycle in the US. Model fit estimations show strong statistical equilibrium before the crisis. Deviations from the equilibrium start to arise between 2004 and 2006, and the first half of the crisis period (2007–June 2008) represents the disequilibrium in the housing market. It is worth mentioning that QRSE model is a statistical equilibrium model, and it might not be able to produce powerful results for the out-of-equilibrium cases such as housing market collapse because forces that keep the system in the statistical equilibrium might not work as well during the crash. In our case, self-organizing dynamics of the system seem to be weakened during the crisis; however, they were still strong enough to be measured in this study. The system seems to start recovering during the second half of the collapse (July 2008–December 2009). After the crisis, the market returns back to statistical equilibrium between 2010 and 2015. Positive average house price growth rates above early pre-crisis period between 2013 and 2015 could be signaling a new housing boom cycle which might necessitate further analysis but is outside of the scope of this study.

Based on our analysis, we argue that increasing average house price growth rates before the housing market collapse created an optimistic environment in a way that weakening responsiveness of individuals’ action on house price growth rate movements (higher *T* compared to crash period) before the collapse is one of the reasons that caused system to build up a period of housing boom/bubble. This may be because glamorous expectations for the future of housing market deteriorated individuals’ capability of processing the market signals. Individuals’ responsiveness reached its maximum during the crisis when the collapse showed itself as a fact rather than just being a possibility.

In addition to weak responsiveness of individuals to market outcomes (higher *T*) between 2004–2006, strong willingness of individuals to sell/buy in the same period seems to represent “wrong (unfulfilled)” expectations which leads to the speculative market behaviors before the crisis. Along with a weakened negative feedback mechanism between actions and house price changes (lower β), strong willingness to transact in the market seems to aggravate further increases in house price growth rates triggering the housing bubble during the pre-crisis periods, although the probability of selling (supply) was higher than the probability of buying (demand). This period, therefore, seems to create a market punishment for the buyers as they kept buying at a “too high prices" when the prices were appreciating at an increasing rate.

Between 2007–2009, even though the feedback mechanism recovered during the first half of the crisis (2007–June 2008) (higher β), the market could not escape from the collapse due to the fact that high responsiveness and deteriorated willingness of selling to declining price growth rates (lower *T* and “unfulfilled” expectations—broadening gap between μ and ξ) prevented selling actions to take place. As a result, the forces keeping the system in statistical equilibrium became unsustainable, and the system-dynamics collapsed leading to dis-equilibrium in the market between 2007 and 2008. Finally, after the crash, average actual growth rate of the market and estimated parameters start to return to their pre-crisis values revealing a recovery of statistical equilibrium after the collapse.

Overall, this study is the first application of the QRSE models on the US housing markets in which we are able to capture and explain different characteristics and behaviors throughout the most recent boom-bust cycle by distinguishing out-of-equilibrium from times of market equilibrium. Modeling highly complex social systems such as housing markets is difficult because their dynamics evolve through the interactions among a large number of heterogeneous individuals under the influence of each others’ decisions and possible external impacts (e.g., policy changes, natural events etc.). This is mostly because market interactions and individuals actions often are not observable to the researcher and the parameter space necessary to model them has high dimensionality, which is not possible to fully account. Moreover, factors such as the lack of high quality data to represent unique non-repetitive social events and the computational inabilities make it even harder. Given the existing difficulties confronted in the literature, we present a simple-alternative method which has a high potential to improve modeling techniques of complex social systems for future economic research problems.

A further extension of the QRSE housing market model can include other economically important variables, such as interest rates, mortgage rates, credit and loan options and debt-to-GDP ratios of different regions—which play significant roles in individual’s buying/selling behavior in order to provide a more economically detailed analysis of housing market dynamics.

One difficulty with QRSE models, on the other hand, is that they require discretization of the data in the form of coarse-grained bins in order to recover statistical equilibrium joint distribution of action *A*, and outcome *x*. Estimation of the model parameters may be affected by the bin size chosen by the modeler. This topic is of interest for future research.

## Figures and Tables

**Table 1:** Estimation results of parameters and related variables based on case-shiller index (vs. FMHPI).

	ID	KL	μ	*T*	β	δ	γ	ξ	Marg. Action Prob.	
		Div.							marg(*a*)	marg($\bar{a}$)
2000-1 & 2003-12	0.96	0.04	−1.07	1.82	4.41	0.26	−2.01	0.39	0.68	0.32
	(0.99)	(0.01)	(−1.64)	(0.94)	(10.12)	(0.28)	(−8.07)	(0.30)	(0.88)	(0.12)
2004-1 & 2006-12	0.98	0.02	−0.62	1.47	1.49	0.44	−0.54	0.35	0.64	0.36
	(0.99)	(0.01)	(−4.7)	(2.5)	(10.02)	(0.37)	(−7.9)	(0.41)	(0.88)	(0.12)
2007-1 & 2008-6	0.97	0.03	1.36	1.25	6.16	1.62	6.07	−1.83	0.09	0.91
	(0.99)	(0.01)	(2.79)	(0.93)	(13.3)	(0.56)	(12.3)	(−0.55)	(0.04)	(0.96)
2008-7 & 2009-12	0.96	0.04	0.52	1.29	0.76	0.75	0.29	−0.47	0.36	0.64
	(0.99)	(0.01)	(−0.35)	(0.86)	(1.68)	(0.31)	(0.36)	(−0.42)	(0.49)	(0.51)
2010-1 & 2012-12	0.99	0.01	−0.6	0.7	0.93	0.49	−0.09	−0.15	0.60	0.40
	(0.99)	(0.01)	(−5.17)	(1.95)	(10.71)	(−0.10)	(−8.83)	(−0.17)	(0.92)	(0.08)
2013-1 & 2015-12	0.97	0.03	−0.83	1.74	3.15	0.36	−1.57	0.57	0.68	0.32
	(0.99)	(0.01)	(−4.09)	(2.05)	(11.13)	(0.34)	(−9.02)	(0.37)	(0.89)	(0.11)

Note: ID represents the information distinguishability measure, which is a measure of closeness between the estimated and observed marginal distributions, Kullback Leibler Divergence is KL Div. (See Appendix C for more details on ID measure and KL Divergence), and the predicted marginal action probabilities of selling and buying are marg(*a*) and marg($\bar{a}$).

## Appendix A. Data

Freddie Mac House Price monthly index, FMHPI, which is available for DC area and 367 Metropolitan statistical areas (MSAs) throughout the US, and the second one is monthly S&P Case-Shiller Index for 20 largest US metropolitan cities called as “Superstar” cities in the literature [39]. Both data sets employ the repeat sales method of index calculation. Compared with S&P Case-Shiller Home Price index, FMHPI has a larger sample size, and covers more geographic areas. FMHPI is based on transactions involving on single family detached and town-home properties purchased by Fannie Mae or Freddie Mac, and is a weighted index, meaning that it measures average price changes in repeat sales or refinancing on the same properties. The S&P index is calculated monthly using a 3 month-moving average algorithm for the data on single-family housing stocks that have sold at least twice in order to capture the appreciation in the values of each sales unit. The most important difference between the two is that the S&P Case-Shiller index is value-weighted which means that price trends for more expensive houses have greater influence on estimated price changes than other homes, whereas FMHPI’s purchase only index weights price trends equally for all properties.

## Appendix B. The Maximum Entropy Program of the QRSE Model for the Housing Market

This section presents the solution to the maximum entropy program, which is used to predict the marginal frequencies, f[x], subject to three constraints; the constraint on the difference between the weighted conditional expected outcomes, Equation (4), a constraint on the mean value of the outcome, ∫f[x]xdx=ξ, and the assumption that the conditional action is a logit function with parameters μ and *T*.

Incorporating the assumption of quantal response, constraint on the difference between the weighted conditional expected outcomes, Equation (4) with the conditional action constraint takes a more compact form which is a hyperbolic tangent function that is:

$$\begin{array}{cc} f[sell]E[x|sell]-f[buy]E[x|buy] & =\int (f[sell|x]f[x]-f[buy|x])f[x])xdx\\ & =\int \left(\frac{1}{1+e^{-}\frac{\left(x-\mu \right)}{T}}-\frac{e^{-}\frac{\left(x-\mu \right)}{T}}{1+e^{-}\frac{\left(x-\mu \right)}{T}}\right)f\left[x\right]xdx\\ & =\int Tanh\left[\frac{\left(x-\mu \right)}{2T}\right]f\left[x\right]xdx\leq \delta \end{array}$$

Since the data on actions are not available, the entropy of the joint distribution can be written in terms of the marginal distribution f[x], as:

$$H=-\int f\left[x\right]Log\left[f\left[x\right]\right]dx+\int H_{\mu ,T}\left[x\right]dx$$

where $H_{\mu ,T}$ is the binary entropy function:

$$H_{\mu ,T}\left[x\right]=-\left(\int f\left[x\right]Log\left[\frac{1}{1+e^{\frac{-(x-\mu )}{T}}}\right]+\int f\left[x\right]Log\left[\frac{e^{\frac{-(x-\mu )}{T}}}{1+e^{\frac{-(x-\mu )}{T}}}\right]\right)$$

Therefore, the maximum entropy program that determines the marginal distribution f[x] given the parameters T,μ,δ,ξ can be expressed as:

$$\begin{array}{cc} \underset{f[x]\geq 0}{\mathrm{Max}} & -\int [f\left[x\right]Log\left[f\left[x\right]\right]dx+\int f\left[x\right]H_{\mu ,T}\left[x\right]dx\\ \mathrm{subject to} & \int f[x]dx=1\\ & \int f[x]xdx=\xi \\ & \int Tanh\left[\frac{\left(x-\mu \right)}{2T}\right]f\left[x\right]xdx\leq \delta \end{array}$$

The Lagrangian to this maxent program, hence is:

$$\begin{array}{cc} L[f[x],\lambda ,\beta ] & =-\int [f\left[x\right]Log\left[f\left[x\right]\right]dx+\int f\left[x\right]H_{\mu ,T}\left[x\right]dx-\lambda \left(\int f[x]dx-1\right)\\ & -\gamma \left(\int f[x]xdx-\xi \right)-\beta \left(\int Tanh\left[\frac{\left(x-\mu \right)}{2T}\right]f\left[x\right]xdx-\delta \right) \end{array}$$

The associated first order conditions for maximizing entropy of the joint frequencies and conditional frequency require:

$$\frac{\partial L}{\partial f[x]}=-Log\left[f\left[x\right]\right]-1-\lambda +H_{\mu ,T}\left[x\right]-\gamma \mu -\beta Tanh\left[\frac{\left(x-\mu \right)}{2T}\right]x=0$$

Solving for f[x] gives the maximum entropy (recovered) marginal distribution $\hat{f}\left[x\right]$ with normalization factor:

$$\hat{f}\left[x\right]=\frac{e^{H_{\mu ,T}\left[x\right]}e^{-\gamma x}e^{\beta Tanh[\frac{x-\mu }{2T}]x}}{\int e^{H_{\mu ,T}\left[x\right]}e^{-\gamma x}e^{\beta Tanh[\frac{x-\mu }{2T}]x}dx}$$

## Appendix C. Posterior Probability Estimates

As mentioned above, predicted marginal distribution $\hat{f}\left[x\right]$ from Equation (5) is a Kernel to the maximum entropy program, and together with the parameters T,μ,β,γ, provides a multinomial distribution for the model. In a Bayesian sense, observed marginal distribution, $\bar{f}\left[x\right]$ is just a sample distribution from the multinomial distribution. We use the Kullback-Leibler divergence as an approximation to the log posterior probability for the multinomial model since it allows us to make posterior inferences about the parameter estimations (See [10] for more details).

Kullback-Leibler divergence measures the discrepancy between the observed marginal frequencies, $\bar{f}\left[x\right]$ and predicted marginal frequencies $\hat{f}\left[x;T,\mu ,\beta ,\gamma \right]$ inferred from the maximum entropy Kernel as:

(A1) $$D_{KL}\left[\hat{f}\left[x\right],\bar{f}[x]\right]=\sum \hat{f}\left[x\right]Log[\frac{\hat{f}\left[x\right]}{\bar{f}\left[x\right]}$$

As a result, KL divergence provides us with a tool to compare the observed marginal frequency distribution with the predicted marginal distribution. If $\hat{f}\left[x\right]=\bar{f}\left[x\right]$, $D_{KL}$ becomes zero indicating that two distribution are the same. Therefore, smaller the KL divergence is closer the observed distribution to the predicted one and better the fit.

Model parameters T,μ,β,γ are estimated jointly by minimizing the $D_{KL}$ function between the observed and inferred marginal frequencies. To measure the closeness of the model fit, we used information distinguishability criteria (ID) introduced by Soofi and Retzer [40]. ID measure shows approximately how much of the informational content of the observed frequencies to be captured from the results of the maximum entropy program.

Finally, to make inferences about the posterior parameters and their credible intervals, conditional distributions of each parameter, holding all others at their maximum posterior probability estimates $\bar{\Omega }$, (where the model parameters are Ω={T,μ,β,γ}), are calculated.
